# Inflated Impact Factors? The True Impact of Evolutionary Papers in Non-Evolutionary Journals

**DOI:** 10.1371/journal.pone.0000999

**Published:** 2007-10-03

**Authors:** Erik Postma

**Affiliations:** School of Biological, Earth and Environmental Sciences, University of New South Wales, Sydney, Australia; University of Exeter, United Kingdom

## Abstract

Amongst the numerous problems associated with the use of impact factors as a measure of quality are the systematic differences in impact factors that exist among scientific fields. While in theory this can be circumvented by limiting comparisons to journals within the same field, for a diverse and multidisciplinary field like evolutionary biology, in which the majority of papers are published in journals that publish both evolutionary and non-evolutionary papers, this is impossible. However, a journal's overall impact factor may well be a poor predictor for the impact of its evolutionary papers. The extremely high impact factors of some multidisciplinary journals, for example, are by many believed to be driven mostly by publications from other fields. Despite plenty of speculation, however, we know as yet very little about the true impact of evolutionary papers in journals not specifically classified as evolutionary. Here I present, for a wide range of journals, an analysis of the number of evolutionary papers they publish and their average impact. I show that there are large differences in impact among evolutionary and non-evolutionary papers within journals; while the impact of evolutionary papers published in multidisciplinary journals is substantially overestimated by their overall impact factor, the impact of evolutionary papers in many of the more specialized, non-evolutionary journals is significantly underestimated. This suggests that, for evolutionary biologists, publishing in high-impact multidisciplinary journals should not receive as much weight as it does now, while evolutionary papers in more narrowly defined journals are currently undervalued. Importantly, however, their ranking remains largely unaffected. While journal impact factors may thus indeed provide a meaningful *qualitative* measure of impact, a fair *quantitative* comparison requires a more sophisticated journal classification system, together with multiple field-specific impact statistics per journal.

## Introduction

Despite the fact that most scientists, funding organisations, promotion committees and journal editors are very much aware of the numerous problems associated with the use of journal impact factors as a measure of scientific quality or even impact [1]–[3], impact factors continue to be amongst the most commonly used measures of journal quality, and thereby of individual papers and their authors. Consequently, researchers take the impact factor of a journal into account when deciding where to submit their work for publication, while journal editors may have the impact factor of their journal in mind when deciding which manuscripts to accept for publication [3].

One of the main problems associated with the use of impact factors as an objective measure of either quality or impact are the large and systematic differences that exist among different scientific disciplines, with impact factors of evolutionary and ecological journals being on the low end of the spectrum [4], [5]. It is these systematic differences among scientific disciplines, which are unrelated to the quality or the size of the field, that make it impossible to directly compare impact factors of journals from different fields [4]–[6].

Indeed, for its annual Journal Citation Reports (JCR), ISI Scientific categorises journals by subject, and journals typically advertise not only their impact factor, but also their ranking within their subject category. Unfortunately however, in a diverse and multidisciplinary field like evolutionary biology, these categories, or any similarly non-hierarchical classification system in which journals can only be a member of a few categories at most for that matter, is far from satisfactory. For example, of the about 5000 articles published between 1996 and 2006 with the term “sexual selection” in either the title, keywords or abstract, only one-fifth was published in journals that are classified by ISI Scientific under ‘*Evolutionary Biology*’ (542 of which were published in Journal of Evolutionary Biology or Evolution), while 423 were published in Proceedings of the Royal Society B (classified under *Biology*), and 392 in Animal Behaviour (classified under *Zoology* and *Behavioural Sciences*). Consequently, we simply cannot avoid making comparisons among journals from a wide range of subject categories, including *Evolutionary Biology*, but also, for example, *Zoology, Ornithology* and *Genetics & Heredity.*

The problem is particularly obvious when comparing impact factors of biological journals on the one hand, and those journals that are classified under *Multidisciplinary Sciences*, which by their very nature publish articles from a wide range of scientific fields. Indeed, there is a general feeling that the extremely high impact factors of multidisciplinary journals like Nature and Science are largely driven by the non-evolutionary and/or non-biological papers they publish [also see e.g. 7]. If this was indeed true, this would imply that, at least for an evolutionary biologist, publishing in these journals is currently overvalued. On the other hand, however, the impact factor of journals that are narrower in focus, and in particular of those limited to a particular taxon, may well underestimate the impact of the evolutionary papers they publish. In other words, what is the true impact of evolutionary papers in non-evolutionary journals?

## Results

**Figure 1:**
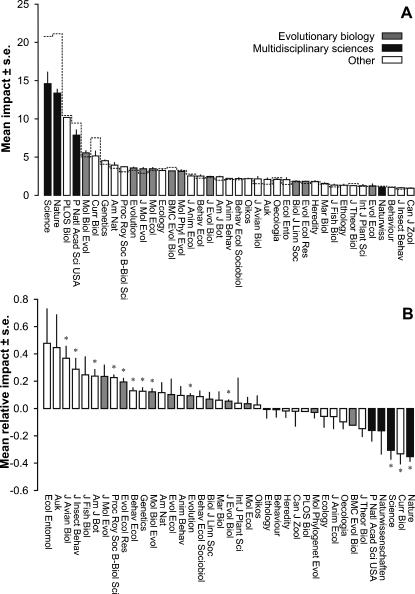
The impact of evolutionary papers in evolutionary and non-evolutionary journals. A) The mean absolute impact of evolutionary papers for 39 journals from a range of different categories. The dotted line gives their overall impact. B) The impact of evolutionary articles relative to the impact of the average article for each of these journals. Relative impacts marked with an asterisk are significantly different from zero at the 5% level.

Not surprisingly, there are highly significant differences among journals in the impact of the evolutionary papers they publish, as well as in their overall impact (Figure 1A). More interestingly, however, the size and/or direction of the difference between these two measures of impact varies among journals, as is indicated by the significant interaction between interaction between Evolutionary vs. Overall impact and Journal in Table 1. In other words, the overall impact of a journal does not necessarily provide a good predictor of the impact of the evolutionary papers it publishes.

**Table 1:** Differences between the average impact of the evolutionary papers a journal publishes and its overall impact factor.

	F	DF	P
*Among journals*			
Journal	231.8	38	<0.001
Year	4.86	4	0.001
*Within journals*			
Evolutionary vs. Overall impact	23.9	1	<0.001
Evolutionary vs. Overall impact×Year	2.14	4	0.079
Evolutionary vs. Overall impact×Journal	19.9	38	<0.001
Error		142	

As indicated by the significant Evolutionary vs. Overall impact × Journal interaction, the size and/or direction of the difference in impact between the evolutionary papers a journal publishes and its overall impact factor varies significantly among journals.

Indeed, Figure 1B shows clearly that for many journals there are large and often significant differences between the impact of the evolutionary articles a journal publishes and a journal's overall impact. While the impact of evolutionary articles published in journals classified under *Multidisciplinary Sciences* are typically between 15 to 30% lower than the impact of the average paper that is published in these journals, evolutionary articles in many of the more specialized, but not explicitly evolutionary, journals are up to 40% percent higher.

Interestingly, also the relative impact of ‘evolutionary’ articles (i.e. an article with one of the ten evolutionary keywords in title, abstract or keywords) published in Evolution and Journal of Evolutionary Biology is significantly greater than zero (although less than 10%). This implies that the ten keywords used to classify an article as ‘evolutionary’ (see Materials and Methods for more details) may not be representative of the whole field of evolutionary biology. To further test for differences among fields within evolutionary biology, we can compare the impact of papers on, for example, sexual selection, natural selection or speciation. Indeed, we find that, at least for papers on sexual selection and natural selection, there are significant differences among journals over and above the differences among journals in the impact of evolutionary papers in general (Table 2). In other words, those journals in which papers on sexual selection have a relatively high impact are not necessarily the same journals in which papers on natural selection have a relatively high impact.

**Table 2:** Differences in the impact of articles on A) sexual selection, B) natural selection, and C) speciation, relative to the impact of evolutionary papers as a whole.

		F	DF	P
**A**	Sexual selection vs. Evolutionary	10.1	1	0.002
	Sexual selection vs. Evolutionary×Year	1.64	4	0.17
	Sexual selection vs. Evolutionary×Journal	3.45	38	<0.001
	Error		135	
**B**	Natural selection vs. Evolutionary	3.24	1	0.074
	Natural selection vs. Evolutionary×Year	0.35	4	0.85
	Natural selection vs. Evolutionary×Journal	3.18	38	<0.001
	Error		132	
**C**	Speciation vs. Evolutionary	2.33	1	0.13
	Speciation vs. Evolutionary×Year	0.91	4	0.46
	Speciation vs. Evolutionary×Journal	0.86	38	0.70
	Error		125	

There are significant differences among journals in the impact of articles on sexual selection and natural selection, but not on speciation, after accounting for differences in the impact of evolutionary articles as a whole. Most importantly, the size and direction of these differences varies across journals. To improve clarity, only within-journal effects are presented.

**Figure 2:**
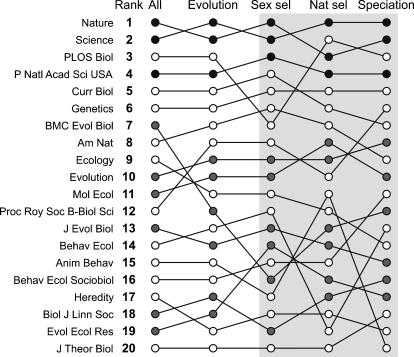
The ranking of journals across (sub-) disciplines. To improve clarity, journals with a very low number of publications between 1996 and 2005 in any of the three sub-disciplines were excluded. As in Figure 1, journals with black dots are classified as *Multidsciplinary Sciences*, journals with grey dots are classified under *Evolutionary Biology*, and journals classified under any other subject category are indicated with an open dot.

Nevertheless, while the above analyses unequivocally show that the overall impact of a journal can substantially under- or overestimate the impact of the evolutionary papers that it publishes, their overall ranking remains remarkably unaffected. A brief look at Figure 1A shows that, compared to the average Evolution paper, evolutionary papers published in Nature or Science do still attract three to four times as many citations in the first two years after their publication. Also if we look at their ranking in more detail, we find that limiting ourselves to the impact of evolutionary papers has in fact only very little effect on a journal's relative ranking, and both rankings are strongly correlated across journals (r = 0.96, P<0.001), particularly for the journals with a relatively high overall impact (Figure 2).

## Discussion

Despite their many inherent problems and limitations, impact factors continue to be used by funding agencies and employers to assess scientists, and this is unlikely to change any time soon. This is at least to some extend justified as impact factors do eliminate some of the bias of other citations statistics, which are more sensitive to the number of papers a journal publishes each year, or the age of a journal. Furthermore, although it may by no means provide a complete measure of impact or quality, a high impact factor, and thus a high average citation rate (in the first years after publication) is by all means a good thing, and a-priori it thus seems reasonable to value a high impact journal over a journal with a lower impact. Although the impact of evolutionary articles published in journals like Nature, Science and PNAS is relatively low compared to the impact of other papers that are published in those journals, which implies that evolutionary papers in any of these journals should maybe not receive as much weight as they do currently, they do indeed have by far the highest impact. Based on this, these journals can indeed be considered to be the most important journals within the field of evolutionary biology.

Evolutionary papers are published in a wide range of journals, most of which are not classified under *Evolutionary biology*. Here I showed that, within these non-evolutionary journals, there are systematic differences in impact among evolutionary and non-evolutionary papers, and to some extent even among different subjects within evolutionary biology. Consequently, quantitative comparisons of journals on the basis of a single impact statistic are fraught with problems for a field like evolutionary biology. If we want to be able to make meaningful comparisons among journals, we will need a more detailed and sophisticated system in which individual papers, rather than journals, are classified into subject categories. Furthermore, these categories should have a hierarchical structure in which papers can be a member of many different categories. For example, a paper on bird behaviour would be a member of *Ornithology* and *Behavioural Sciences*, but automatically also of *Zoology* and *Biology*. Provided the number of papers is sufficiently large, this allows us to calculate separate impact statistics for each of these categories, resulting in multiple, subject-specific impact statistics per journal. This would not only allow for fairer comparisons among journals and researchers, it would also aid authors in maximising the impact of their research, while it would not ‘punish’ editors of high-impact journals for publishing evolutionary or ecological papers.

While I have here focussed on the impact of evolutionary papers in non-evolutionary journals, the approach outlined here can easily be applied to other areas of research and to other journals. This will provide more insight into the generality of the patterns described here, and the extent to which journal impact factors provide biased estimates of impact, either up- or downward. This will help us to interpret variation in journal impact factors among journals and fields, which will hopefully contribute to a fairer assessment of the quality of publications, individual researchers and departments.

## Materials and Methods

### Database

All data were obtained using Thomson Scientific's Web of Science (WoS) (http://isiknowledge.com) in May 2007. The analyses presented here are based on papers published between 1996 and 2005 inclusive only. In an attempt to limit the analyses to research articles only (and exclude e.g. reviews and editorials), only data for publications that are classified in WoS as ‘Article’ were used.

### What is an evolutionary paper?

To objectively and efficiently classify a paper as ‘evolutionary’, independently of the subject category of the journal in which it was published, I compiled a set of keywords that covers a large part of the field of evolutionary biology. I used the 2006 issues of Evolution and Journal of Evolutionary Biology, assuming that papers published in these two journals can be considered to be representative of the field of evolutionary biology. I exported for all articles published in 2006 both the keywords as they are provided by the authors, as well as the KeywordsPlus added by ISI. I then reduced these to those keywords that occur in both journals, and then combined them (while accounting for differences in the total number of keywords between the two journals).

This provides a list of the most common keywords in these two journals, and presumably in evolutionary biology. As some of the most common keywords are not very specific or informative (e.g. evolution, selection, adaptation or body size), these were excluded. This left a list of the following 10 keywords (in order of their relative frequency): 1. Sexual selection; 2. Speciation; 3. Natural selection; 4. Inbreeding depression; 5. Reproductive isolation; 6. Phenotypic plasticity; 7. Gene flow; 8. Phylogeny; 9. Local adaptation; 10. Mate choice. These ten keywords covered about two-thirds of all articles published in Evolution and Journal of Evolutionary Biology.

I subsequently performed searches using all ten keywords (in quotation marks and separated by ‘or’), which gives what I will refer to as evolutionary papers. Additionally, I used the first three keywords separately (which returns papers that I assume are on sexual selection, natural selection, and speciation).

### Calculating impact

I then calculated the impact of a wide range of journals with sufficient papers of an evolutionary nature (see Figure 1), both on the basis of all articles published, and on the basis of those containing any of the above evolutionary keywords.

Impact was calculated in a manner similar to how the impact factors for the JCR are calculated. I thus divided the number of citations to those publications that appeared in a given journal in the two preceding years by the total number of papers published in this journal during these two years. The impact of a journal for 2006 is thus calculated as:
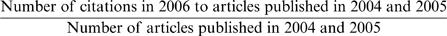



Note that there are two reasons why the overall impact of a journal as it is calculated here (based on all published ‘articles’) is generally lower than the JCR impact factors. First, ISI counts citations to all types of publications (incl. editorials, book reviews etc.) for the numerator, while it only counts papers considered to be citable (articles and reviews) for the denominator. Here on the other hand, both numerator and denominator are based only on those publications that are classified as ‘article’. Second, to obtain the number of citations, I used the ‘Citation analysis’ option in WoS, rather than a ‘Cited reference search’. Although the latter often (but not always) returns more citations as it counts also those citations that are partly incorrect and for example include the wrong year or volume, a ‘Cited reference search’ can not be restricted to a particular keyword, which is essential for this analysis. On the whole, these two factors explain why the average impact of a particular journal based on all articles is generally lower than the ISI impact factor. Nevertheless, both measures of impact are strongly correlated (r = 0.99), as are the journal rankings based on the ISI impact factor and on the measure of overall impact as employed here (r = 0.97).

### Analyses

For each journal I calculated its overall impact, using the number of publications and citations for all articles published, as well as the impact of the evolutionary papers only. Additionally, I calculated the impact of articles on (1) *sexual selection*, (2) *speciation* and (3) *natural selection* (the three most common keywords in Evolution and Journal of Evolutionary Biology) separately. I did this for five years, namely 1998, 2000, 2002, 2004 and 2006, which provides five independent estimates of a journal's impact. To obtain the mean impact for a journal, I averaged across these five periods, while weighing for the number of papers published in each two-year period used in the denominator.

Repeated measures ANOVA's with journal and year as factors where uses to test for significant differences between the impact of evolutionary papers and the overall impact of a journal, all within journals, and in particular whether these differences vary in size and/or direction across journals. Using the same approach, I tested for differences among journals in the impact of papers on natural selection, sexual selection and speciation, over and above the differences among journals in the impact of evolutionary papers as a whole.

Finally, to visualise these differences, relative impact was calculated as (impact evolutionary papers–overall impact)/(overall impact) for each period, and subsequently averaged across the five periods. One-sample t-tests were used to test whether a journal's mean relative impact was significantly different from zero.

## References

[1] Seglen PO (1997). Why the impact factor of journals should not be used for evaluating research.. British Medical Journal.

[2] Garfield E (1996). How can impact factors be improved?. British Medical Journal.

[3] (2006). The Impact Factor Game.. PLoS Medicine.

[4] Kokko H, Sutherland WJ (1999). What do impact factors tell us?. Trends in Ecology & Evolution.

[5] Statzner B (1995). Scale effects on impact factors of scientific journals - Ecology compared to other fields.. Oikos.

[6] Metcalfe NB (1995). Journal impact factors.. Nature.

[7] (2005). Not-so-deep impact.. Nature.

